# Gene expression profiling in C57BL/6J and A/J mouse inbred strains reveals gene networks specific for brain regions independent of genetic background

**DOI:** 10.1186/1471-2164-11-20

**Published:** 2010-01-11

**Authors:** Simone de Jong S, Tova F Fuller, Esther Janson, Eric Strengman, Steve Horvath, Martien JH Kas, Roel A Ophoff

**Affiliations:** 1 Department of Medical Genetics and Rudolf Magnus Institute of Neuroscience, University Medical Center Utrecht, Universiteitsweg 100, 3584 CG Utrecht, The Netherlands; 2 Department of Human Genetics, David Geffen School of Medicine, University of California, Los Angeles, California 90095, USA; 3 Department of Biostatistics, School of Public Health, University of California, Los Angeles, California 90095, USA; 4 Department of Neuroscience and Pharmacology, Rudolf Magnus Institute of Neuroscience, University Medical Center Utrecht, Universiteitsweg 100, 3584 CG Utrecht, The Netherlands; 5 Center for Neurobehavioral Genetics, University of California, Los Angeles, CA 90095, USA

## Abstract

**Background:**

We performed gene expression profiling of the amygdala and hippocampus taken from inbred mouse strains C57BL/6J and A/J. The selected brain areas are implicated in neurobehavioral traits while these mouse strains are known to differ widely in behavior. Consequently, we hypothesized that comparing gene expression profiles for specific brain regions in these strains might provide insight into the molecular mechanisms of human neuropsychiatric traits. We performed a whole-genome gene expression experiment and applied a systems biology approach using weighted gene co-expression network analysis.

**Results:**

We were able to identify modules of co-expressed genes that distinguish a strain or brain region. Analysis of the networks that are most informative for hippocampus and amygdala revealed enrichment in neurologically, genetically and psychologically related pathways. Close examination of the strain-specific gene expression profiles, however, revealed no functional relevance but a significant enrichment of single nucleotide polymorphisms in the probe sequences used for array hybridization. This artifact was not observed for the modules of co-expressed genes that distinguish amygdala and hippocampus.

**Conclusions:**

The brain-region specific modules were found to be independent of genetic background and are therefore likely to represent biologically relevant molecular networks that can be studied to complement our knowledge about pathways in neuropsychiatric disease.

## Background

Genome-wide gene expression profiling has been used to aid in the discovery of genes involved in human diseases and to discriminate between disease subtypes. This approach has already been successfully applied in cancer and obesity research [1,2], but similar approaches have not yet been widely applied to human neuropsychiatric traits. An important issue concerning expression profiling in these disorders in man is the lack of the most relevant tissue, i.e. the brain, for study. Except for limited numbers of post-mortem samples, there is no easy access to human neuronal tissue for expression studies [3].

Mouse models, however, have long been used to study neuropsychiatric traits. There is consensus that symptoms of disorders such as major depression and anxiety can be studied by observing behavior in inbred strains [4,5]. Moreover, mouse brain tissue is accessible and most brain regions are highly conserved between mouse and human [6,7]. Regional gene expression in the brain has been shown to be conserved between non-human primates, rodents and man, making it possible to study mice in relation to human neurobiology [8,9].

This study focuses on two brain regions, the hippocampus and amygdala, because of their importance in neural behavior and psychiatric disease. These areas are both part of the limbic system and known to be heavily interconnected. Hippocampal function has been extensively studied and it has been shown to be a key structure in learning and memory processes [10]. The amygdala is important in processing fear and anxiety and is also thought to be involved in associative learning and memory. Because of its small size and complicated anatomy the amygdala has been less well studied in man. The structure consists of about 10 different subnuclei with distinct roles for the input and output of information [11]. Both brain regions have been implicated in neuropsychiatric disorders such as schizophrenia and depression, as well as epilepsy, but there is increasing evidence that they might play differential roles as well [12,13].

Using the mouse as an animal model is advantageous for the study of neurobehavioral traits because the genetic make-up of these inbred strains is known and they have been extensively phenotyped for more than two decades. This makes it possible to select strains most divergent for the (behavioral) trait of interest and to search for underlying genetic factors [14]. A recent study estimated that there are ~8 million single nucleotide polymorphisms (SNPs) between classical mouse inbred strains, which results in an estimated strain diversity of 1 SNP every 250-300 base pairs [15].

Although gene expression carries information about biological state and environment, a number of studies have shown that up to 85% of the measured transcripts in human lymphocytes are significantly heritable [16,17]. This suggests that gene expression in general is under strong genetic control. An association analysis of expression levels with SNPs and genomic copy number variations (CNVs) in human lymphoblastoid cell lines was performed and showed that SNPs and CNVs capture 83.6% and 17.7% of the total detected genetic variation in gene expression, respectively [18]. Expression levels are shown to be more variable among individuals than among the populations they belong to, as studied in *fundulus *fish and man [17,19,20]. Differences in allele frequencies seem to explain most of the variation in transcript levels between human populations [21].

To explore gene expression differences specific to a strain and/or brain region, we studied the amygdala and hippocampus brain tissues of mouse inbred strains A/J and C57BL/6J. These strains have been consistently shown to differ in behavioral, physiological and developmental processes. For example, studies have reported that A/J show more anxiety-like behaviors and are less social towards other mice [22]. A/J is also known to have lower motor activity levels [23] and be more resistant to developing epilepsy [24] than C57BL/6J.

Although they are heavily interconnected, the amygdala and hippocampus perform separate functions in memory and emotion (see earlier). The amygdala functions mostly through GABA-ergic transmission, while the hippocampus uses excitatory connections [6]. Here we explore whether behavioral differences between the mouse strains and functional specificity of the two brain regions are reflected in differential gene expression profiles. It is plausible that differences in expression profiles represent a combination of genetic and functional variation. Considering the involvement of the hippocampus and amygdala in neurobehavioral symptoms in both mouse and man, the results from our mouse analysis may shed new light on pathways underlying neuropsychiatric traits in humans as well.

Since we were interested in finding co-expression modules that relate to amygdala and hippocampus in the different mouse strains, we used gene co-expression network analysis. Gene co-expression network methods have been successfully applied in a variety of different settings [8,25-29]. This systems biology analysis method starts out by defining clusters of co-expressed genes (called 'modules') which may represent molecular networks involved in a common biological pathway. Genes that are highly connected within these groups are thought to drive the modules and are considered to be 'hub genes'. As described below, we identified large co-expression modules that are significantly associated with differences between mouse strains and brain regions. We found that the observed strain-specific modules in fact reflect hybridization artifacts but, strikingly, two large modules distinguish brain regions irrespective of genetic background. Detailed functional enrichment analyses revealed an overrepresentation of genes involved in neurological and psychological disorders, in addition to nervous system function and development.

## Results

### Data preprocessing

After background correction, expression arrays were transformed and normalized according to the Lumi procedure [30]. Genes were then filtered based on detection values generated by BeadStudio^©^. The detection *p*-value threshold was set at 0.01, leaving 13,627 of the 24,620 probes on the microarrays for analysis. Hierarchical clustering using all probe information yielded no sample outliers.

### Network reconstruction

A gene co-expression network was constructed using amygdala and hippocampus tissue from both A/J and C57BL/6J strains (total N = 35). For computational reasons, only the 5,000 most variable probes (across all samples) were used for the network construction. To construct a weighted co-expression network based on the matrix of pairwise Pearson correlation coefficients, we used a soft thresholding approach by raising each correction to a fixed power. We used the criterion described by Zhang and Horvath to choose the power (beta = 8) [29]. One advantage of soft thresholding is that it leads to networks that are highly robust with respect to different soft-thresholds (beta). In particular, our findings remained virtually unchanged for different choices of beta. Soft thresholding resulted in a 5,000 × 5,000 dimensional weighted adjacency matrix containing pairwise connection strengths. Next, a connectivity measure (*k*) per probe was calculated by summing the connection strengths with other network genes. Since module genes tend to be highly connected, we restricted the module detection analysis to genes that had relatively high connectivity. We chose a connectivity threshold of 0.1, which resulted in 2,795 probes for the module detection analyses.

### Module detection

We define co-expression modules as branches of a hierarchical clustering tree. Specifically, we used average linkage hierarchical clustering with the topological overlap similarity measure to define a cluster tree. The topological overlap is a robust measure of interconnectedness, which keeps track of shared patterns of connection strengths [29,31-33].

**Figure 1:**
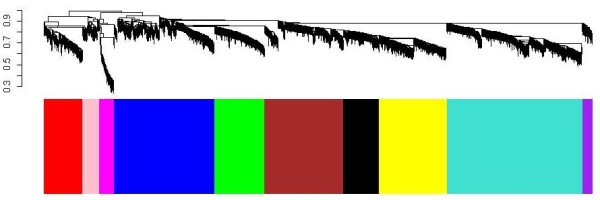
**Network construction identifies distinct modules of co-expressed genes using hippocampus and amygdala samples of A/J and C57BL/6J mice (n = 35)**. The dendrogram was produced by average linkage hierarchical clustering of genes using topological overlap. Modules of co-expressed genes were assigned colors corresponding to the branches indicated by the horizontal bar beneath the dendrogram.

For branch cutting (module detection) we used the dynamic branch-cutting algorithm implemented in the dynamicTreeCut and WGCNA (weighted gene co-expression network analysis) R library [26,34]. Each module (or branch) is assigned a unique color label which is visualized in the color band underneath the cluster tree (see Figure 1). We found 10 modules, each of which contained at least 50 probes. Our module detection method followed the standard WGCNA approach that has been successfully used in multiple applications [8,25,26,29,35]. Since our modules are large and distinct, we expect them to be found by many alternative detection methods. Average gene expression levels of all modules range from 8.52 to 9.51.

### Modules related to strain and brain region

To identify modules that are related to the sample traits of interest - here mouse strain status (A/J versus C57BL/6J) and brain region (amygdala versus hippocampus) - we correlated the sample traits with a representative measure of each module. To define a representative module expression profile (referred to as the module eigengene), we summarized the (standardized) gene expression profiles of the module by their first principal component. The module eigengene can be considered a weighted average of the module gene expression profiles.

The correlation between the eigengene module and the sample trait of interest (e.g. brain region status) is referred to as eigengene significance. A standard correlation test can be used to assess the statistical significance (*p*-value) of the eigengene significance. Our module-based analysis has a major advantage over a standard differential gene expression analysis: since it only relates a handful of modules to the sample trait, the module-based analysis circumvents the multiple comparison problem that plagues standard gene-based analyses (that relate thousands of probes to the sample trait). We found a highly significant correlation between the Magenta module eigengene (based on 76 probes) and strain status (*r *= 0.99, *p *< 1.0e-29). This shows that the Magenta module is comprised of genes that are highly differentially expressed between the two strains. Strikingly, we found that brain region status (amygdala versus hippocampus) was highly correlated with both the Red module (*r *= -0.99, *p *= 1.8e-29, 198 probes,) and the Pink module (*r *= -0.81, *p *= 4.4e-09, 84 probes) eigengenes. All of these *p*-values remain highly significant even after carrying out the most stringent multiple comparison adjustment (Bonferroni correction) for the number of modules.

**Figure 2:**
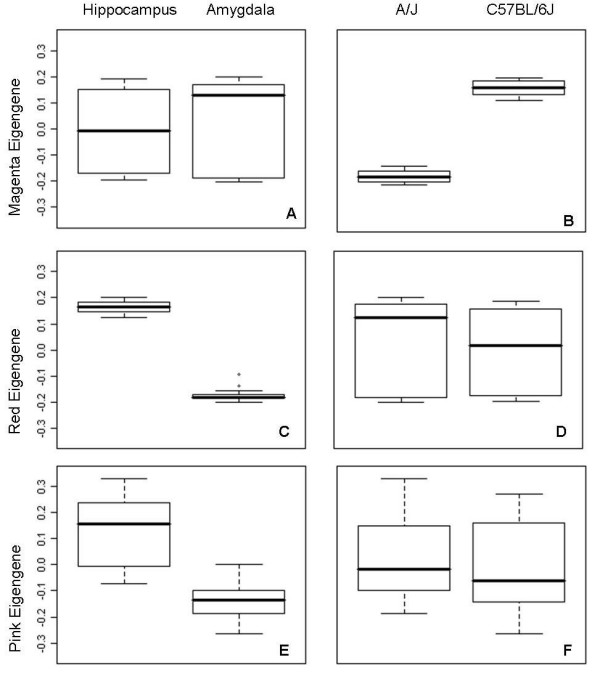
**Module eigengene values can separate samples based on mouse strain or brain region**. The module eigengene is a single representative expression profile for each sample, based on the first principal component of that module. The box plots show that the Magenta eigengene values separate samples perfectly into an A/J and C57BL/6J group (b) but not brain region (a). The Red module eigengenes creates groups of amygdala and hippocampus samples (c), but does not separate between strains (d). The Pink module eigengene differentiates between brain regions as well, but to a lesser extent (e and f).

Figure 2a-f and Additional File 1 show the eigengene expression values of the Magenta, Red and Pink modules. The eigengene comparison showed that the Magenta module completely separates the inbred mouse strains, while the Pink and Red modules distinguish the amygdala and hippocampus independent of strain origin. The Red module is most informative in distinguishing the brain regions amygdala and hippocampus.

**Figure 3:**
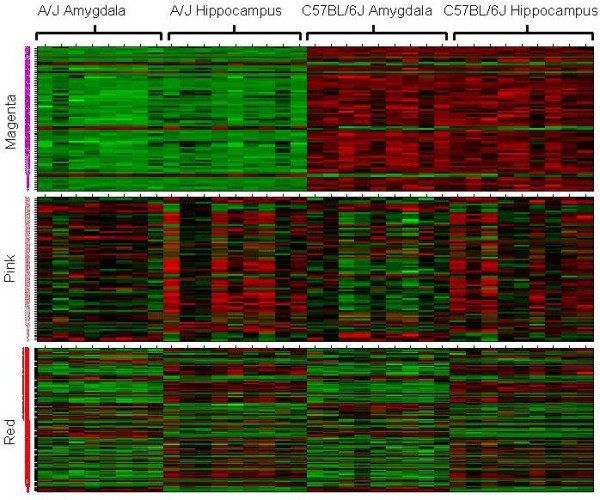
**Gene expression heat map for significant modules**. The rows correspond to genes and the columns to samples, with samples groups indicated at the top. Genes colored green are under-expressed, while red indicates over-expression. Expression in the Magenta module (top panel) is lower in A/J compared to C57BL/6J for almost all probes. Both up- and down-regulated probes compose the Pink (middle panel) and Red (bottom panel) modules when comparing amygdala and hippocampus.

A heat map of the Magenta module revealed a distinct pattern of overall decreased expression levels in A/J compared to C57BL/6J (Figure 3). The heat map of the Pink and Red modules showed a more random distribution of increased and decreased gene expression levels on comparing amygdala and hippocampus. The full data set with the Magenta, Red and Pink module gene content is available online (see Additional File 2). In addition, the correlation between the eigengenes for the Red, Pink and Magenta modules with expression values of all probes (i.e. 'module membership') detected on the expression arrays are given in Additional File 3.

### Preservation of modules

To assess the preservation of the modules within each strain and brain region, multiple post-hoc analyses were performed. Samples were either separated by strain (containing amygdalar and hippocampus samples, *n *= 17 A/J samples, *n *= 18 C57BL/6J samples) or brain region (containing samples from both A/J and C57BL/6J origin, *n *= 17 amygdalar samples, *n *= 18 hippocampal samples). Networks of co-expressed genes were constructed for these different groups using the color coding from the previous module definition (using all samples from both strains).

To assess the existence of the brain region specific modules in separate strains, networks of co-expressed genes were constructed for each strain separately (using both amygdalar and hippocampus samples per strain) and using the color code of the previous module definition (using all samples of both strains). Modules are preserved when genes with the same color continue to cluster together based on their topological measure in the separate networks. Visual inspection of the hierarchical clustering trees shows that this is the case for the Red and Pink modules, but not for the Magenta module (see Additional File 4). The preservation of module eigengene significance of the Red and Pink modules was used to validate these gene networks as enriched with genes differentially expressed between amygdala and hippocampus independent of origin of strain. The module eigengene correlation for the Red module was significant in both the A/J (*r *= 0.99, *p *= 2.4e-16) and C57BL/6J (*r *= 0.99, *p *= 5.4e-14) networks. The Pink module also remained significant on comparing amygdala and hippocampus in A/J and C57BL/6J (*r *= 0.88, *p *= 7.7e-05 and *r *= 0.82, *p *= 3.8e-05).

To assess the preservation of the Red and Pink modules, we used a connectivity-based module preservation measure described in [8,25,35]. Specifically, for a given module, this preservation measure is defined as the correlation of the intramodular connectivity between the two strains. Thus, the higher the value of the module preservation measure, the better preserved is the module structure (its connectivity pattern) between the two strains. For the Pink and Red modules, we found a highly significant module preservation measure (*r *= 0.53 *p *< 10e-03) and (*r *= 0.51 (*p *< 10e-03), respectively, which shows that these modules are highly preserved between the two strains.

The same post-hoc analyses were performed to assess preservation of the modules of interest in networks constructed using either amygdalar or hippocampus samples (from both strains) with probes colored according to the initial network (constructed on all samples from both strains). Visual inspection of the hierarchical clustering trees of the amygdalar and hippocampus samples separately shows preservation of the Magenta module, but not of the Red or Pink modules (see Additional File 4). Magenta module eigengene significance remained high for strain differences in the networks created with hippocampal samples (*r *= 0.99, *p *= 1.1e-14) and amygdala (*r *= 0.99, *p *= 1.3e-14) separately. We found that the Magenta module was highly preserved between amygdalar and hippocampal networks (*r *= 0.80, *p *< 10e-03).

### Polymorphic SNPs in module genes and probes

To investigate whether genetic variation between the two inbred strains could affect module detection, we collected SNP data from genes represented in the Magenta, Pink and Red modules (Table 1). SNPs were counted in target genes with a 10 kb region around the gene location. A comparison between the three modules showed that the Magenta module contained the smallest number of total SNPs relative to the number of interrogated base pairs compared to the Pink (*p *< 2.2e-16) and Red (*p *< 2.2e-16) modules; the Pink module contained significantly more total SNPs than the Red module (*p *< 2.2e-16). However, the Magenta module, which was enriched with genes that are differentially expressed between A/J and C57BL/6J, contained significantly more non-synonymous coding SNPs than those found in the Pink (*p *= 2.6e-07) and Red (*p *= 3e-03) modules; the Pink and Red modules did not differ in their numbers of non-synonymous coding SNPs (*p *= 0.04).

**Table 1:** SNP counts within target genes in significant modules

	Total bp checked	Total SNPs	% of bp	Non-synonymous coding SNPs	% of SNPs found
**Magenta**	21,218,367	7,128	0.03	59	0.83
**Pink**	8,637,477	8,193	0.09	19	0.23
**Red**	15,909,861	10,464	0.07	49	0.47

The first column refers to the number of base pairs checked within a module. To include possible regulatory regions, a margin of 10 kb before and after each gene was used. The second column contains the total number of SNPs found, and the third column shows this number relative to the number of base pairs checked. Non-synonymous coding SNPs are represented in column four, and column five shows the percentage of non-synonymous coding SNPs compared to the total number of SNPs found.

When checking probe sequences for known SNPs between A/J and C57BL/6J, the Magenta module was composed of significantly more SNP-containing probes (32 of 76 probes) than the Red module (3 out of 198 probes, *p *< 2.2e-16) and the Pink module (1 out of 84 probes, *p *= 1.3e-11). The number of probes containing SNPs did not differ significantly between the Red and Pink modules. In addition, sequencing of eleven of the top probes of the Magenta modules without known SNP revealed that 91% (10 of the 11) contain a polymorphism between C57BL/6J and A/J, with the C57BL/6J genomic sequence being identical to the probe sequence in all cases. Results are given in Additional File 5.

In order to better understand the high frequency of sequence variation in probe regions of the Magenta module genes, we also investigated whether the target genes of the probes in the Magenta module with and without a SNP were *cis*- or *trans*-regulated genes. We consulted the publicly available WTCCC heterogeneous stock database containing expression quantitative trait loci (eQTL) in hippocampus for this purpose [36]. This data was also collected using the Illumina platform and differentiates between real *cis*-effects and possible false *cis*-acting eQTLs resulting from probes with and without a SNP in the sequence. Of the 32 target genes with probes resident in the Magenta module and containing a SNP, more possible false *cis-*acting eQTLs were detected in the WTCCC database than the 44 remaining probes of the Magenta module (13 out of 32 vs. 7 out of 44 probes, *p *= 0.02). Moreover, probes in the Magenta module without known SNPs were significantly enriched with real *cis*-acting eQTLs compared to the probe sequences containing a SNP (16 out of 44 vs. 3 out of 32, *p *< 0.01).

### Gene ontology enrichment analysis

**Figure 4:**
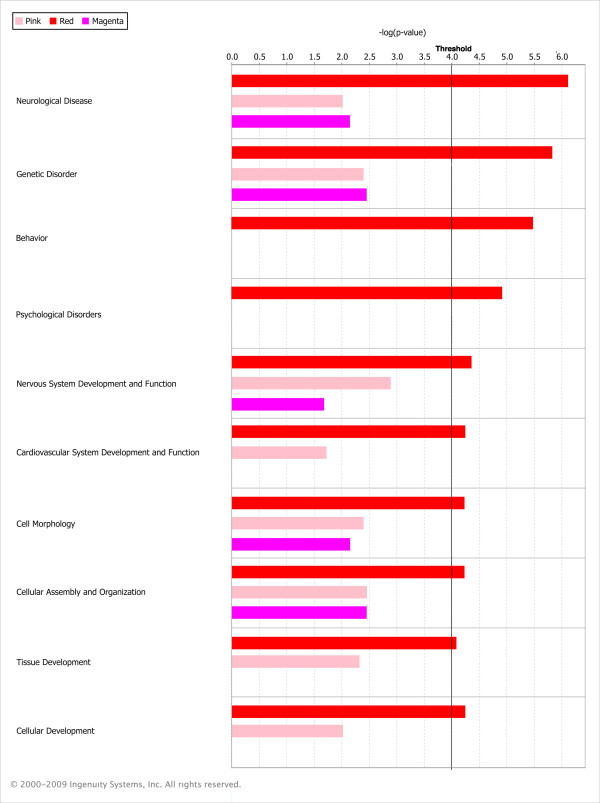
**Color-coded bar plot depicting Ingenuity Pathway Analysis results**. We used Ingenuity Pathways Analysis (Ingenuity^® ^Systems, http://www.ingenuity.com) to study the ontology of genes in the Red, Pink and Magenta modules. Results for categories enriched by at least one module at the *p *< 10e-04 level are depicted in the color-coded bar plot.

**Figure 5:**
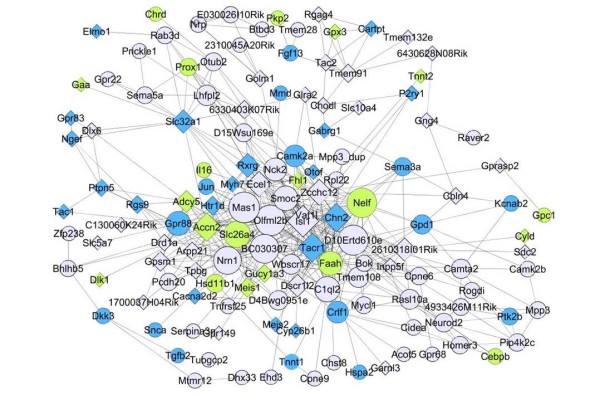
**Visual representation of connections of genes in Red module**. This figure shows target genes of the probes in the Red module with the strongest connections only (*r *> 0.92). Circles (○) denote genes with increased expression levels in hippocampus and diamonds (◊) represent genes with increased expression levels in the amygdala. Node size is related to the number of connections of that particular gene; a highly connected gene (i.e. 'hub gene') is therefore larger than genes with fewer connections. Nodes are colored blue when they appear in the Ingenuity categories Neurological Disease, Genetic Disorder and Psychological Disorder. Green nodes represent the genes unique to the Genetic Disorder category. Edge length is not related to correlation strength.

We used Ingenuity Pathways Analysis (Ingenuity^® ^Systems, http://www.ingenuity.com) to study the ontology of genes in the Pink and Red modules - these modules were enriched for genes that are differentially expressed between amygdala and hippocampus. For the Red module (165 out of 198 genes are known) the top-3 overrepresented subcategories within 'diseases and disorders' were Neurological Disease (*p *= 7.6e-7 - 3.3e-2), Genetic Disorder ((1.5e-6 - 2.7e-2) and Psychological Disorder (*p *= 1.2e-5 - 1.5e-2). The category 'physiological system development and function' yielded the following top-3: Behavior (*p *= 3.3e-6 - 2.2e-2), Nervous System Development and Function (*p *= 4.4e-5 - 3.3e-2) and Cardiovascular System Development and Function (*p *= 5.7e-5 - 2.9e-2). The Pink module (72 out of 84 genes were in the Ingenuity Pathways Knowledge Base) yielded no significant results using this threshold. The Magenta module (68 out of 76 genes are known) had significant overrepresentation of genes in the 'Diseases and Disorders' subcategory Dermatological diseases and conditions (6.6e-4 - 7.1e-3). Results for categories enriched by at least one module at the *p *< 10e-04 level are shown in the color-coded bar plot in Figure 4. The complete results from the Ingenuity analysis are provided in Additional File 6. A visual representation of the network for the Red module is given in Figure 5, highlighting the strongest connections between hub genes and the involvement of the genes in different functional pathways.

## Discussion

In this study, we applied systems biology methods to identify modules of co-expressed genes highlighting differences between the hippocampus and amygdala in two mouse inbred strains, A/J and C57BL/6J. Rather than generating a list of differentially expressed genes, this approach reconstructs networks of genes with related expression profiles that are thought to represent biological meaningful correlations. Reconstruction of these modules is performed in an unbiased fashion, independent of strain or tissue origin. These results may provide new insights or complement our existing knowledge on brain region-specific molecular pathways. Since the hippocampus and amygdala are largely conserved between man and mouse, and are thought to play an important role in behavior, emotion and cognition, studying these brain regions may aid our understanding of mechanisms underlying neuropsychiatric traits.

We observed differential expression between A/J and C57BL/6J mouse inbred strains and between the amygdala versus hippocampus brain regions (Figure 3). Further analysis demonstrated that differences in gene expression profiles between brain tissues were independent of strain origin (Figure 2, Additional File 4). While we provide evidence that the observed strain-specific differences are likely the result of hybridization artifacts (i.e. Magenta module), we postulate that networks enriched with genes that are differentially expressed between amygdala and hippocampus (i.e. Pink and Red modules) represent biological relevant molecular pathways. A limitation of our study is that both hippocampus and amygdala are not homogeneous (see for example [37,38]). Future research could aim to further dissect these regions (e.g. by laser capture) so that more homogenous sub-regions could be studied. Our study provides a more global assessment of differential expression between these large brain structures thereby ignoring the regional substructures within each of the brain regions.

We observed that brain region-specific effects can be detected independent of strain origin. Genetic variation is thought to be a major regulator of gene expression [16,18,39]; however, the differential expression profiles between the amygdala and hippocampus as observed in our study seem to represent natural variation driven by tissue-specific biological processes instead. The Red module especially differentiates between brain region samples, without any interference from genetic background (Figure 2c-d). Previous studies investigating differences in brain regions also found that regional expression could be detected independent of strain. Whether genetic or tissue-specific effects have a stronger influence on gene expression is still under debate [40-44]. The results from our study suggest that the magnitude of the effect of strain and brain region is about the same, as module significance and eigengene values are comparable. However, since the module separating strains may not contain actual strain effects but be due to a hybridization artifact, it is likely that the effect of genetic strain differences is smaller in reality than the functional differences between tissues. Interaction effects between strain and brain region of all 13,627 probes were also assessed (see Additional File 7). This analysis revealed only 8 genes (*Arsj*, *Baiap2l1*, *Fgf10, Myoc, Krt9, Lyd, 4930511J11Rik, Lypd1*) of which differential expression between amygdala and hippocampus was dependent on strain. Of these, *Fgf10 *and *4930511J11Rik *belonged to the Red module. These small numbers suggests that interaction effects between strain and brain region are very limited.

The current dataset provides a comparison between two brain region tissues only and is therefore somewhat limited in its perspective. While Red and Pink modules clearly separate amygdala and hippocampus in these two strains, the functions represented in these modules may extend to other (brain) regions as well. When we compared our findings with spinal cord data from the same individuals, only two of the ten modules were preserved in spinal cord (i.e. Magenta and Turquoise). As in the amygdala and hippocampus, the Magenta module again strongly differentiated between strains in spinal cord, suggesting that the SNP artifact in the probe sequences is driving the strain-specific differences throughout different neuronal tissues. The Red and Pink module differentiating between amygdala and hippocampus, however, are not conserved in spinal cord and are therefore more likely to be amygdala or hippocampus specific.

The Magenta module is enriched for genes differentially expressed between strains independent of brain region. The module eigengene of this group of target genes was able to fully distinguish A/J and C57BL/6J samples (Figure 2a-b). Closer examination revealed that a significant number of probe sequences of genes in the Magenta module contain nucleotide variants (SNPs) that differ between these two strains. The Illumina expression arrays used in this study were developed using the C57BL/6J as the reference strain with probes optimally designed for the C57BL/6J genome. If there are SNP variants between strains within a probe sequence it is expected that C57BL/6J hybridization will be more effective. This prediction coincides with the systematically lower expression levels of probes in the Magenta module in strain A/J (Figure 3). When the network is re-constructed after removing genes in the magenta module that were known to contain SNPs, the remaining magenta colored genes still fall into the same cluster. This module also still differentiates between strains. Therefore, it is possible that these probes represent real strain differences. However, resequencing of the probe sequences not containing a known SNP revealed that there are many unknown strain-specific polymorphisms in probe regions that may affect gene expression measurements between strains.

The issue of SNP variants in probe regions causing hybridization artifacts has been described before when short (25-mer) cDNA probes were used [45,46], and more recently for long (60-mer) oligonucleotide probes [47]. In addition, it was found that mismatches do affect hybridization intensity, depending on the position of the SNP. By studying the enrichment of modules with regard to SNP-containing probes, our module-based analysis was able to detect this technical artifact. Further, we found that significantly increased numbers of SNP variants at the probe regions were also observed at loci with strong *cis*-effects in an *e*QTL analysis of the WTCCC heterogeneous stock database.

A standard gene-based analysis could have easily missed this systematic bias. Co-expression modules may represent technical artifacts, tissue contaminations, or other biologically uninteresting perturbations. This is why functional enrichment analyses, module preservation studies across array platforms, and other forms of validation are required to verify that co-expression modules are indeed biologically meaningful. In our data, we found that a functional enrichment analysis for known gene ontologies did not find any significant findings for the Magenta module (which arose due to hybridization artifacts). We conclude that careful analysis of probe regions is warranted in an expression array study, especially when only a limited number of (parental) inbred strains are involved. We expect that our findings also apply to transgenic mouse models in which part of the genomic sequence may still be from a different genetic background.

The hybridization artifact is not present in the modules found to be significantly enriched with genes differentially expressed between the amygdala and hippocampus samples in both strains, i.e. the Red and Pink modules (Figures 2, 3, Additional File 2). Available databases were used to validate our brain region specific results in silico (Allen Brain Atlas; http://www.brain-map.org/). In both modules, only ~10% of the probes disagreed in expression direction of amygdala and hippocampus, thereby largely validating current brain-region specific findings. Previous studies also found some expression differences between these structures, although they are more similar to each other than most brain regions [41,42]. Functional examination of the genes represented in these two modules was performed using Ingenuity (Ingenuity^® ^Systems, http://www.ingenuity.com). This analysis revealed that the Pink module did not contain any known pathways. The module eigengene of this network of co-expressed genes was also shown to be less discriminative between samples from the different brain areas than the Red module. However, the Red module was shown to be enriched with the categories of Neurological Disease, Genetic Disorder and Psychological Disorder, as well as Behavior and Nervous System Processes.

A major advantage of WGCNA is that its module detection does not make use not make use of any prior gene ontology information. This allows the expression data to speak for themselves without biasing the analysis. But gene ontology information is very valuable for determining what is known about the modules. While some modules may be highly enriched with genes of a given gene ontology there is no perfect agreement between gene ontology information and module membership. As we show, modules may arise due to non-biological variation. But biologically important modules must not always be enriched with known gene ontologies [8]. While the Ingenuity database is very extensive, GO analyses remain limited by incomplete gene ontology annotation. Module membership and network connectivity may point to a relationship between genes and pathways that was hitherto unknown. Figure 5 indicates genes that are known to fall within these categories, as well as genes that are found to be in the same module based on co-expression profiles but have not been implicated in these pathways before. Therefore, this network approach may provide insights into new candidates in already known processes.

The connectivity of the genes in this module is an indication of their interactivity with other genes in the same module. As can be seen in the graphical representation of the network of the Red module (Figure 5), several genes in enriched pathways are highly connected, while others are less related to other genes. For example *Tacr1 (tachykinin receptor 1) *is a centrally located hub gene in the Red module and appears in all the above-mentioned categories determined by Ingenuity to be significantly enriched. This gene is more highly expressed in the amygdala compared to the hippocampus in our dataset. This receptor for the substance P is located in the central nucleus of the amygdala. Substance P is a neuropeptide related to pain and it has also been implicated in a wide range of behaviors including learning and memory [48], motivational processes, and anxiety [49]. Other genes in the Red module are also of added value since they may complement and/or interconnect our knowledge of pathways without having a known, large effect on neuropsychiatric traits directly. For example, we observed an enrichment of genes involved in transcriptional processes that distinguished between the amygdala and hippocampus; these genes included *Isl1*, *Fhl2*, *Tnfrsf25 *and *Zcchc12*.

Other examples in the Red module support previous findings from the literature. For example, genes involved in the canonical pathway 'GABA-ergic signaling' (*Slc32a1*, *Gabrg1 *and *Gad1*) are closely correlated in the Red module, with increased expression levels in the amygdala. In addition, genes in the subcategory 'neurogenesis of nervous system development' (*Nrn1*, *Cpne6*, *Dlx6*, *Enc1*, *Fgf13*, *Isl1*, *Nelf*, *Neurod2*, *Olfm1*, *Sema3a*, *Sema3f*, *Sema5a*, *Tgfb2*, *Wfs1*) were also found in the same module. Adult neurogenesis takes place predominantly in the hippocampus (and olfactory bulb) [50] and most of these genes (except for *Dlx6*, *Hap1*, *Isl1 *and *Wfs1*) indeed showed an increased expression in the hippocampus [51].

## Conclusions

We report very distinct patterns of gene expression between brain regions independent of genetic variation due to differences between mouse inbred strains. Differential gene expression patterns associated with strain origin were highly enriched with SNP variation within the probe regions. In our study design we found that tissue-specificity is at least as important for variation in gene expression as genetic variation. Functional enrichment studies of modules related to the mouse brain regions implicate known human neurological disease pathways, which suggests that effective mouse models can be developed for human disease.

## Methods

### Animals

For this experiment, male C57BL/6J and A/J mice were used. Initial breeding pairs for these strains were obtained from the Jackson Laboratory and bred in the Rudolf Magnus Institute of Neuroscience animal facility at the UMC Utrecht. All experimental procedures were approved by the ethical committee for animal experimentation of the University Medical Center Utrecht, The Netherlands. All mice used for the experiment were bred under standard conditions (21 ± 1.0°C and 50% humidity) in Macrolon cages (type II-extended, type number: 1284 L.) maintained in a 12-hr light/12-hr dark cycle. Four weeks after birth, mice were weaned and socially housed (2-4 same sex littermates per cage) with ad libitum access to food and water. Mice were sacrificed at 3-4 months of age and their brains were quickly removed, frozen in liquid nitrogen and stored at -80°C. After dissection of selected brain areas we had 17 amygdala samples (8 A/J and 9 C57BL/6J) and 18 hippocampus samples (9 A/J and 9 C57BL/6J).

### Dissection procedure

Brain samples were thawed from -80°C storage to -8°C in cryostat. Coronal sections of 300 μm thickness were taken. Frozen sections were laid down on a cooled steel plate, covered with parafilm and immediately covered with RNAlater^® ^(Ambion, #AM7024). Selected brain regions were punched out using a stainless steel punch needle (1 mm in diameter) filled with RNAlater connected to a syringe. The amygdala were captured in 5 sections starting at -0.58 mm Bregma with one punch taken bilaterally for a total of 10 punches. Hippocampal tissue was taken in 6 sections starting at -2.06 mm Bregma with two punches taken bilaterally in the first three sections, and three in the last three, for a total of 15 punches. Tissue from the left hippocampus was used in the current study.

### RNA isolation

RNAlater was pipetted off the samples. Punches were homogenized using disposable pestels (Fisher Scientific Pellet Stamp, #749521-1500). Phase separation was achieved using 750 μl TRIzol reagent (Invitrogen, #15596-018) and 200 μl chloroform (Merck #8.222.65.1000), after which samples were precipitated in 500 μl isopropanol (Merck #1.0934.2500). DNAse treatment (Qiagen Rnase-Free Dnase Set, #79254) was applied using the manufacturer's protocol, followed by an RNA clean-up procedure (Qiagen RNeasy MinElute columns, #74204). Samples were stored at -80°C. Total RNA concentration was checked using the Nanodrop ND-1000 and RNA quality was checked using Bioanalyzer RNA chips (Agilent Technologies RNA Nano kit, #5067-1511).

### Microarrays

Genome-wide RNA expression profiling was obtained with the Illumina MouseRef-8 V1.1 arrays using Illumina's standard protocol. In short, RNA samples were prepared with the Illumina TotalPrep kit amplification and labeling protocol (Ambion, #IL1791). Amplified and biotinylated cRNA was measured with a ribogreen assay (Invitrogen Quant-it™ Ribogreen, #R11490), and 750 ng of labeled cRNA was then used for array hybridization. Individual mouse samples were hybridized to separate arrays. BeadChips were scanned using an Illumina BeadArray reader. Data is made available at Gene Expression Omnibus (GSE17955; http://www.ncbi.nlm.nih.gov/geo/).

### Statistical analysis

BeadStudio^© ^software version 3.2.3 was used to extract raw data and generate background-corrected gene-expression data. Background correction was performed by subtracting the average value of negative control beads present on the array. To demonstrate that our findings are not dependent on the background correction method, we repeated the analysis with non-background corrected gene expression data. Our conclusions remain unchanged: we find highly similar grouping of the genes and relationships to the phenotypes. Further pre-processing was done using the Lumi package for R [30]. A variance stabilizing transformation was applied to preserve much of the gene-expression variance. Data were normalized using the robust spline normalization method [52]. Chip quality and outlier detection was performed by assessing quality statistics and plots (hierarchical clustering, box plots, density distribution plots, pair-wise correlations) before and after transformation and normalization. Modules of co-expressed genes were identified using weighted gene co-expression network analysis (WGCNA) developed by Zhang and Horvath, implemented in a freely available R packge [26,29]. First, a correlation matrix for these genes was constructed. This matrix was then raised to a power (beta = 8 in this study) to achieve an adjacency matrix holding connection strengths. Connectivity is defined as the sum of connection strengths with the other network genes. A topological overlap measure is calculated based on the number of shared neighbors. A dendrogram is produced by hierarchical clustering of 1 minus the topological overlap; branches of the tree are cut using a dynamic tree cut algorithm to define modules [34]. The module eigengene is the first principal component of a module and can therefore be thought of as an average gene expression value for all genes in a module per sample. Module significance for either a continuous or dichotomous outcome is determined assessing eigengene module significance. The importance of individual genes could be assessed by looking at both gene significance and connectivity measures in the whole network and within modules.

### Polymorphic SNPs in genes and probes

Analyses assessing polymorphisms between mouse inbred strains were performed *in silico*. Gene start and end positions were obtained by entering Illumina identifiers in Biomart http://www.biomart.org, which uses ENSMBL data. To include possible regulatory regions, a margin of 10 kb before and after each gene was used. This region was then entered in the Mouse Phenome Database (NCBI 37, http://phenome.jax.org/pub-cgi/phenome/mpdcgi?rtn=docs/home), comparing SNPs between A/J and C57BL/6J. Total SNPs were counted and the number of synonymous and non-synonymous coding SNPs was recorded. In addition, Illumina 50-mer probe sequences were blasted (NCBI megaBLAST, http://blast.ncbi.nlm.nih.gov/Blast.cgi) against the FASTA sequences, including the flanking sequences (obtained via NCBI SNP 37.1, http://www.ncbi.nlm.nih.gov/sites/entrez, of SNPs in exons or UTR regions. The significance of the differences between SNP counts was tested using a Fisher's exact test for count data with a threshold of *p *< 0.05. Cis effects were checked in the eQTL database generated with the heterogeneous stock of the Welcome Trust Case Control Consortium http://gscan.well.ox.ac.uk/. The significance of the differences between counts was tested using a Fisher's exact test for count data with a threshold of *p *< 0.05. In addition, sequencing of probe sequences not containing a known SNP was performed. Primers were designed in NCBI's Primer BLAST http://www.ncbi.nlm.nih.gov/tools/primer-blast/ to generate a PCR product. Sequencing was performed according to standard protocols on an ABI 3730 (Applied Biosystems) sequencer. A list of probes and primers can be found in Additional File 5.

### Gene ontology analysis

Ontological enrichment for functional categories was studied using Ingenuity Pathways Analysis (Ingenuity^® ^Systems, http://www.ingenuity.com). We obtained a bar plot of functional categories with at least one module enriched at the *p *< 10e-04 level.

## Abbreviations

SNP: Single Nucleotide Polymorphism; CNV: Copy Number Variation; GABA: γ-Aminobutyric acid; WGCNA: Weighted Gene Coexpression Analysis.

## Authors' contributions

SDJ carried out dissections and molecular genetic studies, performed statistical analyses and sequence alignment, interpreted results and drafted manuscript. TFF provided statistical assistance carried out gene ontology analyses. EJ and ES provided technical assistance. SH provided oversight over statistical and gene ontology analyses. MJHK and RAO conceived of the study and participated in its design and coordination. All authors participated in completion of the manuscript.

## Supplementary Material

Additional file 1 **Eigengene significance for mouse strain and brain region**. The module eigengene is a single representative expression profile for each sample, based on the first principal component of that module. The first bar plot shows that the Magenta module eigengene is significantly correlated with strain. This indicates that this module is significantly enriched for genes differentially expressed between A/J and C57BL/6J. The second barplot shows that the Red and Pink module eigengenes are significantly correlated with brain region. This indicates that these modules are significantly enriched for genes differentially expressed between amygdala and hippocampus. Click here for file

Additional file 2 **Content of the Magenta, Pink, and Red modules**. Tables contain probes in Magenta (table 1), Pink (table 2) and Red (table 3) modules. Illumina probe ID, Gene Symbol, Chromosome and expression differences AJ versus C57BL6/J and hippocampus versus amygdala are given. Asterisks in the Probe ID column denote probes containing a known SNP between A/J and C57BL/6J. The last column denotes the cis-regulated genes according to the WTCCC Heterogeneous stock database http://gscan.well.ox.ac.uk/, with and without known SNPs between included strains. Expression data is transformed (variance stabilizing method) and normalized (robust spline) yielding values comparable to LOG2 values. Click here for file

Additional file 3 **Magenta, Pink and Red module membership for all genes**. Table contains the correlation between the eigengenes (i.e. 'module membership') for the Red, Pink and Magenta modules with expression values for all 13,627 detected genes on Illumina array. Click here for file

Additional file 4 **Network reconstruction on subsets of samples**. Networks constructed per strain or per brain region identify distinct modules of co-expressed genes. Dendrograms were produced by average linkage hierarchical clustering of genes using the topological overlap measure. Modules of co-expressed genes were assigned colors corresponding to the branches indicated by the horizontal bar beneath each dendrogram. The color code of the previous module definition (using all samples of both strains) was used to assess preservation. The Red and Pink module are preserved in networks constructed on just A/J (n = 17) or just C57BL/6J samples (n = 18). The Magenta module is found in the networks constructed on just amygdalar (n = 17) or just hippocampus samples (n = 18). Click here for file

Additional file 5 **Additional polymorphisms in probe sequences**. Sequencing was performed for the probes not containing a known SNP. Probes in the Magenta module showing the highest differential expression between C57BL/6J and A/J were selected. The first and second columns contain the Illumina probe ID and gene symbol of the corresponding target gene. The third column shows the probe sequence for these probes. The forward and reverse primers used are shown in column 4 and 5. The last column indicates whether a SNP (or indel) was found in the probe sequence, which is also indicated in the probe sequence. Click here for file

Additional file 6 **Ingenuity Pathway Analysis for the modules of interest**. Ingenuity pathway results for genes in the Red (table 1), Pink (table 2) and Magenta (table 3) modules. Click here for file

Additional file 7 Strain * brain tissue interaction effects. A linear model analysis was performed to assess possible interaction effects of brain tissue * strain for all 13627 probes. For this, the R package Limma was used, constructing a linear model with interaction term. Significance was assessed using FDR correction. After this correction, 6 genes showed significant interaction (adjusted *p*-value < 0.01). The first three columns of the table contain the Illumina Probe ID, Gene Symbol and chromosome. Column four indicates the module in which the probe was grouped using WGCNA analysis. Linear model statistics are given in column five and six. Click here for file

## References

[B1] Sotiriou C Pusztai L Gene-expression signatures in breast cancer N Engl J Med 2009 360 8 790 800 10.1056/NEJMra0801289 19228622

[B2] Zhu J Zhang B Schadt EE A systems biology approach to drug discovery Adv Genet 2008 60 603 635 full_text 18358334 10.1016/S0065-2660(07)00421-X

[B3] Sequeira A Klempan T Canetti L ffrench-Mullen J Benkelfat C Rouleau GA Turecki G Patterns of gene expression in the limbic system of suicides with and without major depression Mol Psychiatry 2007 12 7 640 655 10.1038/sj.mp.4001969 17353912

[B4] Bourin M Petit-Demouliere B Dhonnchadha BN Hascoet M Animal models of anxiety in mice Fundam Clin Pharmacol 2007 21 567 574 10.1111/j.1472-8206.2007.00526.x 18034657

[B5] El Yacoubi M Vaugeois JM Genetic rodent models of depression Curr Opin Pharmacol 2007 7 1 3 7 10.1016/j.coph.2006.11.002 17169613

[B6] Kandel ER Schwartz JH Jessell TM Principles of Neural Science 2000 4 McGraw-Hill Companies

[B7] West MJ Stereological studies of the hippocampus: a comparison of the hippocampal subdivisions of diverse species including hedgehogs, laboratory rodents, wild mice and men Prog Brain Res 1990 83 13 36 full_text 2203095 10.1016/s0079-6123(08)61238-8

[B8] Oldham MC Horvath S Geschwind DH Conservation and evolution of gene coexpression networks in human and chimpanzee brains Proc Natl Acad Sci USA 2006 103 47 17973 17978 10.1073/pnas.0605938103 17101986 PMC1693857

[B9] Strand AD Aragaki AK Baquet ZC Hodges A Cunningham P Holmans P Jones KR Jones L Kooperberg C Olson JM Conservation of regional gene expression in mouse and human brain PLoS Genet 2007 3 4 e59 10.1371/journal.pgen.0030059 17447843 PMC1853119

[B10] Wittenberg GM Tsien JZ An emerging molecular and cellular framework for memory processing by the hippocampus Trends Neurosci 2002 25 10 501 505 10.1016/S0166-2236(02)02231-2 12220877

[B11] Dolan RJ Emotion, cognition, and behavior Science 2002 298 5596 1191 1194 10.1126/science.1076358 12424363

[B12] Kempermann G Krebs J Fabel K The contribution of failing adult hippocampal neurogenesis to psychiatric disorders Curr Opin Psychiatry 2008 21 3 290 295 10.1097/YCO.0b013e3282fad375 18382230

[B13] Krishnamoorthy ES A differential role for the hippocampus and amygdala in neuropsychiatric disorders J Neurol Neurosurg Psychiatry 2007 78 11 1165 1166 10.1136/jnnp.2006.108084 17940166 PMC2117583

[B14] van Gaalen MM Steckler T Behavioural analysis of four mouse strains in an anxiety test battery Behav Brain Res 2000 115 1 95 106 10.1016/S0166-4328(00)00240-0 10996412

[B15] Frazer KA Eskin E Kang HM Bogue MA Hinds DA Beilharz EJ Gupta RV Montgomery J Morenzoni MM Nilsen GB A sequence-based variation map of 8.27 million SNPs in inbred mouse strains Nature 2007 448 7157 1050 1053 10.1038/nature06067 17660834

[B16] Goring HH Curran JE Johnson MP Dyer TD Charlesworth J Cole SA Jowett JB Abraham LJ Rainwater DL Comuzzie AG Discovery of expression QTLs using large-scale transcriptional profiling in human lymphocytes Nat Genet 2007 39 10 1208 1216 10.1038/ng2119 17873875

[B17] Stranger BE Nica AC Forrest MS Dimas A Bird CP Beazley C Ingle CE Dunning M Flicek P Koller D Population genomics of human gene expression Nat Genet 2007 39 10 1217 1224 10.1038/ng2142 17873874 PMC2683249

[B18] Stranger BE Forrest MS Dunning M Ingle CE Beazley C Thorne N Redon R Bird CP de Grassi A Lee C Relative impact of nucleotide and copy number variation on gene expression phenotypes Science 2007 315 5813 848 853 10.1126/science.1136678 17289997 PMC2665772

[B19] Oleksiak MF Churchill GA Crawford DL Variation in gene expression within and among natural populations Nat Genet 2002 32 2 261 266 10.1038/ng983 12219088

[B20] Storey JD Madeoy J Strout JL Wurfel M Ronald J Akey JM Gene-expression variation within and among human populations Am J Hum Genet 2007 80 3 502 509 10.1086/512017 17273971 PMC1821107

[B21] Spielman RS Bastone LA Burdick JT Morley M Ewens WJ Cheung VG Common genetic variants account for differences in gene expression among ethnic groups Nat Genet 2007 39 2 226 231 10.1038/ng1955 17206142 PMC3005333

[B22] Moy SS Nadler JJ Young NB Perez A Holloway LP Barbaro RP Barbaro JR Wilson LM Threadgill DW Lauder JM Mouse behavioral tasks relevant to autism: phenotypes of 10 inbred strains Behav Brain Res 2007 176 1 4 20 10.1016/j.bbr.2006.07.030 16971002 PMC1857288

[B23] Kas MJ de Mooij-van Malsen JG de Krom M van Gassen KL van Lith HA Olivier B Oppelaar H Hendriks J de Wit M Groot Koerkamp MJ High-resolution genetic mapping of mammalian motor activity levels in mice Genes Brain Behav 2009 8 1 13 22 10.1111/j.1601-183X.2008.00435.x 18721260

[B24] van Gassen KL Hessel EV Ramakers GM Notenboom RG Wolterink-Donselaar IG Brakkee JH Godschalk TC Qiao X Spruijt BM van Nieuwenhuizen O Characterization of febrile seizures and febrile seizure susceptibility in mouse inbred strains Genes Brain Behav 2008 7 5 578 586 10.1111/j.1601-183X.2008.00393.x 18363854

[B25] Ghazalpour A Doss S Zhang B Wang S Plaisier C Castellanos R Brozell A Schadt EE Drake TA Lusis AJ Integrating genetic and network analysis to characterize genes related to mouse weight PLoS Genet 2006 2 8 e130 10.1371/journal.pgen.0020130 16934000 PMC1550283

[B26] Langfelder P Horvath S WGCNA: an R package for weighted correlation network analysis BMC Bioinformatics 2008 9 559 10.1186/1471-2105-9-559 19114008 PMC2631488

[B27] Shieh GS Chen CM Yu CY Huang J Wang WF Lo YC Inferring transcriptional compensation interactions in yeast via stepwise structure equation modeling BMC Bioinformatics 2008 9 134 10.1186/1471-2105-9-134 18312694 PMC2323972

[B28] Stuart JM Segal E Koller D Kim SK A gene-coexpression network for global discovery of conserved genetic modules Science 2003 302 5643 249 255 10.1126/science.1087447 12934013

[B29] Zhang B Horvath S A general framework for weighted gene co-expression network analysis Stat Appl Genet Mol Biol 2005 4 Article 17 10.2202/1544-6115.1128 16646834

[B30] Du P Kibbe WA Lin SM lumi: a pipeline for processing Illumina microarray Bioinformatics 2008 24 13 1547 1548 10.1093/bioinformatics/btn224 18467348

[B31] Li A Horvath S Network neighborhood analysis with the multi-node topological overlap measure Bioinformatics 2007 23 2 222 231 10.1093/bioinformatics/btl581 17110366

[B32] Ravasz E Somera AL Mongru DA Oltvai ZN Barabasi AL Hierarchical organization of modularity in metabolic networks Science 2002 297 5586 1551 1555 10.1126/science.1073374 12202830

[B33] Yip AM Horvath S Gene network interconnectedness and the generalized topological overlap measure BMC Bioinformatics 2007 8 22 10.1186/1471-2105-8-22 17250769 PMC1797055

[B34] Langfelder P Zhang B Horvath S Defining clusters from a hierarchical cluster tree: the Dynamic Tree Cut package for R Bioinformatics 2008 24 5 719 720 10.1093/bioinformatics/btm563 18024473

[B35] Horvath S Zhang B Carlson M Lu KV Zhu S Felciano RM Laurance MF Zhao W Qi S Chen Z Analysis of oncogenic signaling networks in glioblastoma identifies ASPM as a molecular target Proc Natl Acad Sci USA 2006 103 46 17402 17407 10.1073/pnas.0608396103 17090670 PMC1635024

[B36] Valdar W Flint J Mott R Simulating the collaborative cross: power of quantitative trait loci detection and mapping resolution in large sets of recombinant inbred strains of mice Genetics 2006 172 3 1783 1797 10.1534/genetics.104.039313 16361245 PMC1456308

[B37] Zirlinger M Selection and validation of microarray candidate genes from subregions and subnuclei of the brain Methods 2003 31 4 290 300 10.1016/S1046-2023(03)00158-0 14597313

[B38] Zirlinger M Kreiman G Anderson DJ Amygdala-enriched genes identified by microarray technology are restricted to specific amygdaloid subnuclei Proc Natl Acad Sci USA 2001 98 9 5270 5275 10.1073/pnas.091094698 11320257 PMC33199

[B39] Stranger BE Forrest MS Clark AG Minichiello MJ Deutsch S Lyle R Hunt S Kahl B Antonarakis SE Tavare S Genome-wide associations of gene expression variation in humans PLoS Genet 2005 1 6 e78 10.1371/journal.pgen.0010078 16362079 PMC1315281

[B40] Fernandes C Paya-Cano JL Sluyter F D'Souza U Plomin R Schalkwyk LC Hippocampal gene expression profiling across eight mouse inbred strains: towards understanding the molecular basis for behaviour Eur J Neurosci 2004 19 9 2576 2582 10.1111/j.0953-816X.2004.03358.x 15128411

[B41] Nadler JJ Zou F Huang H Moy SS Lauder J Crawley JN Threadgill DW Wright FA Magnuson TR Large-scale gene expression differences across brain regions and inbred strains correlate with a behavioral phenotype Genetics 2006 174 3 1229 1236 10.1534/genetics.106.061481 16980393 PMC1667050

[B42] Sandberg R Yasuda R Pankratz DG Carter TA Del Rio JA Wodicka L Mayford M Lockhart DJ Barlow C Regional and strain-specific gene expression mapping in the adult mouse brain Proc Natl Acad Sci USA 2000 97 20 11038 11043 10.1073/pnas.97.20.11038 11005875 PMC27144

[B43] Zapala MA Hovatta I Ellison JA Wodicka L Del Rio JA Tennant R Tynan W Broide RS Helton R Stoveken BS Adult mouse brain gene expression patterns bear an embryologic imprint Proc Natl Acad Sci USA 2005 102 29 10357 10362 10.1073/pnas.0503357102 16002470 PMC1173363

[B44] Hovatta I Zapala MA Broide RS Schadt EE Libiger O Schork NJ Lockhart DJ Barlow C DNA variation and brain region-specific expression profiles exhibit different relationships between inbred mouse strains: implications for eQTL mapping studies Genome Biol 2007 8 2 R25 10.1186/gb-2007-8-2-r25 17324278 PMC1852412

[B45] Kirst M Caldo R Casati P Tanimoto G Walbot V Wise RP Buckler ES Genetic diversity contribution to errors in short oligonucleotide microarray analysis Plant Biotechnol J 2006 4 5 489 498 17309725 10.1111/j.1467-7652.2006.00198.x

[B46] Sliwerska E Meng F Speed TP Jones EG Bunney WE Akil H Watson SJ Burmeister M SNPs on chips: the hidden genetic code in expression arrays Biol Psychiatry 2007 61 1 13 16 10.1016/j.biopsych.2006.01.023 16690034

[B47] Rennie C Noyes HA Kemp SJ Hulme H Brass A Hoyle DC Strong position-dependent effects of sequence mismatches on signal ratios measured using long oligonucleotide microarrays BMC Genomics 2008 9 317 10.1186/1471-2164-9-317 18598341 PMC2475537

[B48] Kertes E Laszlo K Berta B Lenard L Effects of substance P microinjections into the globus pallidus and central nucleus of amygdala on passive avoidance learning in rats Behav Brain Res 2008 17;198 2 397 403 10.1016/j.bbr.2008.11.021 19071162

[B49] Gadd CA Murtra P De Felipe C Hunt SP Neurokinin-1 receptor-expressing neurons in the amygdala modulate morphine reward and anxiety behaviors in the mouse J Neurosci 2003 23 23 8271 8280 12967989 10.1523/JNEUROSCI.23-23-08271.2003 PMC6740689

[B50] Ming GL Song H Adult neurogenesis in the mammalian central nervous system Annu Rev Neurosci 2005 28 223 250 10.1146/annurev.neuro.28.051804.101459 16022595

[B51] Emilsson V Thorleifsson G Zhang B Leonardson AS Zink F Zhu J Carlson S Helgason A Walters GB Gunnarsdottir S Genetics of gene expression and its effect on disease Nature 2008 452 7186 423 428 10.1038/nature06758 18344981

[B52] Lin SM Du P Huber W Kibbe WA Model-based variance-stabilizing transformation for Illumina microarray data Nucleic Acids Res 2008 36 2 e11 10.1093/nar/gkm1075 18178591 PMC2241869

