# Supplementary figures and images for: Gene expression profiling in C57BL/6J and A/J mouse inbred strains reveals gene networks specific for brain regions independent of genetic background
Source: BMC Genomics. 2010 Jan 11;11:20. doi: 10.1186/1471-2164-11-20 (PMC2823687; doi:10.1186/1471-2164-11-20)

Eigengene Significance for Strain

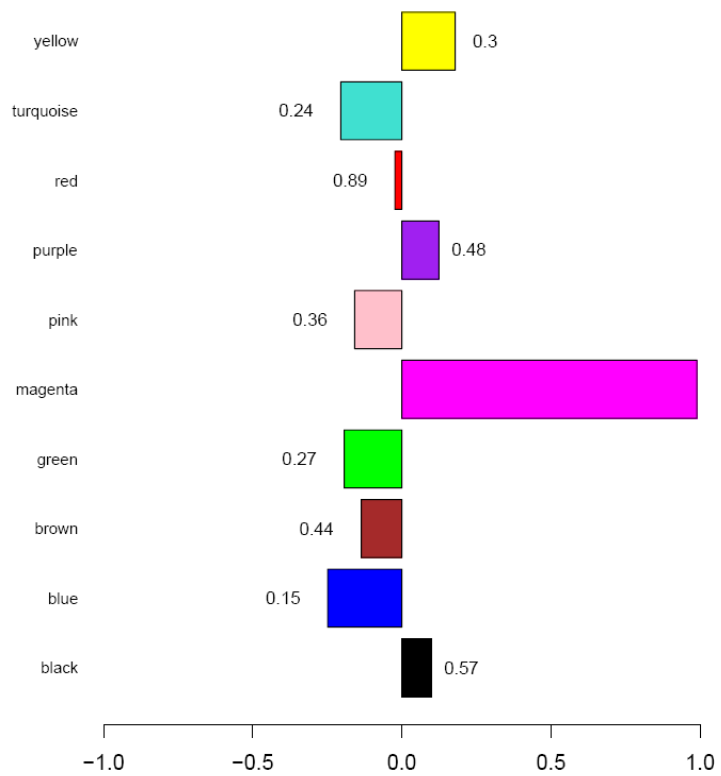

Eigengene Significance for Brain

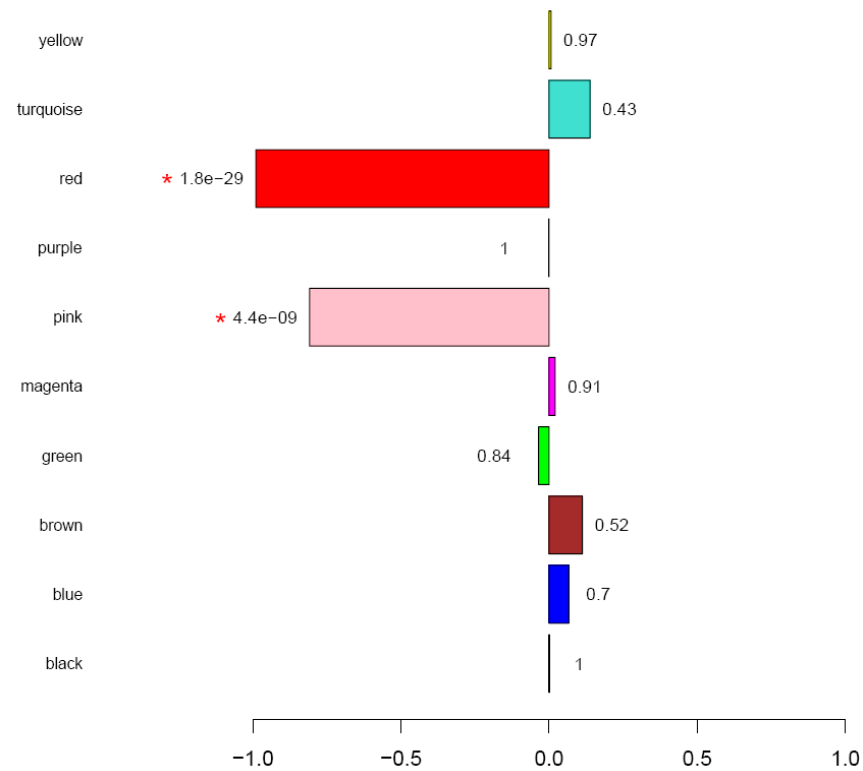

Supplement: Additional file 1 — Eigengene significance for mouse strain and brain region. The module eigengene is a single representative expression profile for each sample, based on the first principal component of that module. The first bar plot shows that the Magenta module eigengene is significantly correlated with strain. This indicates that this module is significantly enriched for genes differentially expressed between A/J and C57BL/6J. The second barplot shows that the Red and Pink module eigengenes are significantly correlated with brain region. This indicates that these modules are significantly enriched for genes differentially expressed between amygdala and hippocampus. [file 1471-2164-11-20-S1.PDF]
