# Supplementary material for: Gene expression profiling in C57BL/6J and A/J mouse inbred strains reveals gene networks specific for brain regions independent of genetic background
Source: BMC Genomics. 2010 Jan 11;11:20. doi: 10.1186/1471-2164-11-20 (PMC2823687; doi:10.1186/1471-2164-11-20)
Supplement: Additional file 4 — Network reconstruction on subsets of samples. Networks constructed per strain or per brain region identify distinct modules of co-expressed genes. Dendrograms were produced by average linkage hierarchical clustering of genes using the topological overlap measure. Modules of co-expressed genes were assigned colors corresponding to the branches indicated by the horizontal bar beneath each dendrogram. The color code of the previous module definition (using all samples of both strains) was used to assess preservation. The Red and Pink module are preserved in networks constructed on just A/J (n = 17) or just C57BL/6J samples (n = 18). The Magenta module is found in the networks constructed on just amygdalar (n = 17) or just hippocampus samples (n = 18). [file 1471-2164-11-20-S4.PDF]

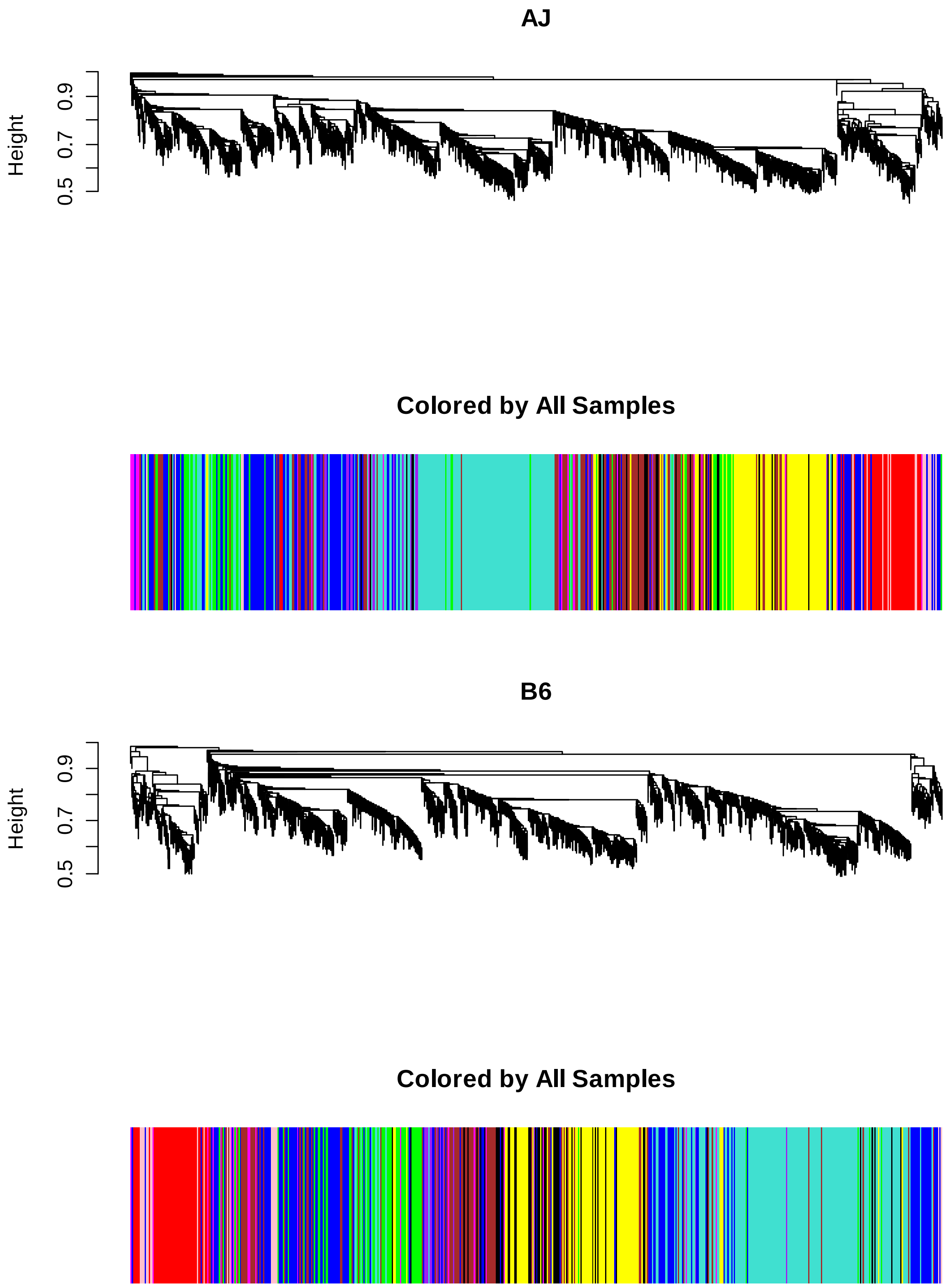
AJ
Height
0.5
0.7
0.9
Colored by All Samples
B6
Height
0.5
0.7
0.9
Colored by All Samples

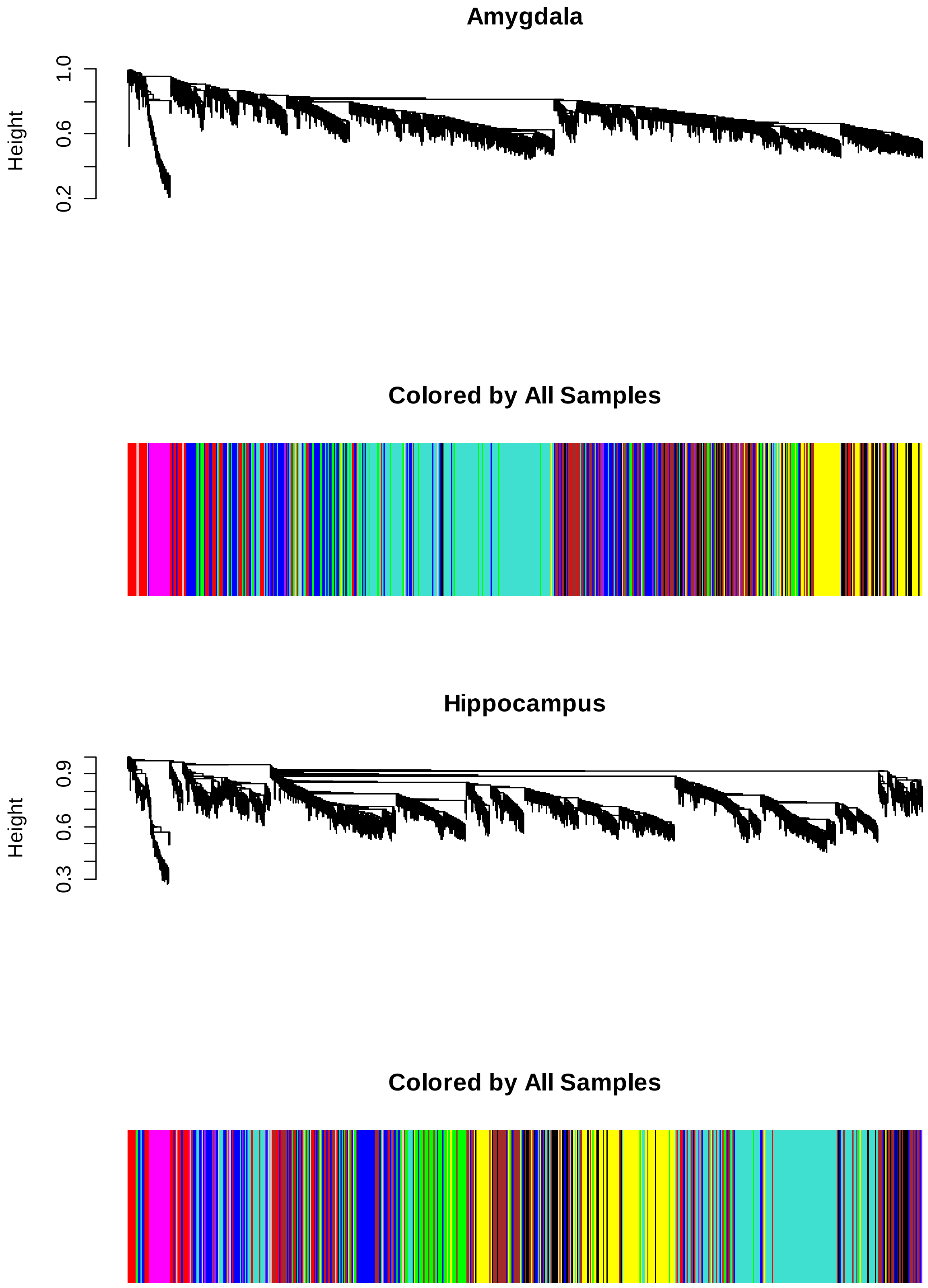
Amygdala
Height
1.0
0.6
0.2
Colored by All Samples
Hippocampus
Height
0.9
0.6
0.3
Colored by All Samples
